# Current Surgical Trends in Carpal Tunnel Syndrome

**DOI:** 10.1055/a-2769-7554

**Published:** 2026-01-30

**Authors:** Sang Hyun Woo, Bong Gyu Choi, Soo Jin Woo, Kwang Hyun Park

**Affiliations:** 1 W Institute for Hand and Reconstructive Microsurgery, W General Hospital, Daegu, South Korea

**Keywords:** carpal tunnel syndrome, median nerve, nerve compression syndromes

## Abstract

This review highlights current surgical approaches for carpal tunnel syndrome (CTS), the most common compressive neuropathy of the upper extremity. Open, mini-open, endoscopic, and emerging minimally invasive techniques are compared in terms of outcomes, complications, pillar pain, and reoperation rates. Surgical indications, anatomical considerations, and management of recalcitrant CTS—including recurrent, persistent, and new-onset symptoms—are discussed. Adjunct procedures such as opponensplasty and flexor synovectomy are reviewed, with emphasis on patient selection, individualized decision-making, and the importance of thorough anatomical knowledge to ensure safe adoption of novel techniques.

## Introduction

Carpal tunnel syndrome (CTS) is the most common compressive neuropathy of the upper extremity. The prevalence of clinically diagnosed CTS is 3.8%, 1 and the mean annual incidence is 360.26 per 100,000 person-years in Korea. 2 Some proponents of conservative management question the value of carpal tunnel release (CTR), particularly because mild-to-moderate CTS can improve with nonsurgical measures such as splinting, oral medications, injections, electrotherapy, manual therapy, and neural gliding exercises. 3 4 However, there is no clear consensus on the optimal nonsurgical regimen, regarding its duration, the number of modalities to use, or the appropriate timing for transitioning to surgery. 5

By contrast, substantial evidence demonstrates that surgical intervention provides clear benefits, offering superior symptom relief and functional improvement at 6 and 12 months postoperatively, and yielding a twofold higher rate of normalization in nerve conduction studies. Even in severe CTS, significant symptomatic improvement is typically observed following CTR. 6 7 8 Nevertheless, surgery is not without risks, and potential complications and side effects must be considered.

Drawing on our experience with over 15,000 CTR procedures at a single institution, we review recent literature including original studies, systematic reviews, and meta-analyses, to highlight current trends in the surgical management of CTS.

This review includes institutional clinical images and case descriptions. Written informed consent for the publication of clinical information and images was obtained from all patients.

## Surgical Indication

The primary objective of CTR is to prevent irreversible injury to the median nerve (MN). Complete division of the flexor retinaculum can relieve symptoms, halt disease progression, and restore hand function while minimizing the risk of complications.

Accurate diagnosis is essential before surgical decision-making. CTS can often be diagnosed on the basis of medical history and physical examination alone. The CTS-6 diagnostic tool, which evaluates numbness in the MN distribution, nocturnal numbness, thenar muscle weakness or atrophy, Tinel sign, Phalen test, and loss of two-point discrimination, considers a score of ≥12 diagnostic for CTS. 9 For differential diagnosis, ultrasonography and electrodiagnostic testing are recommended as validated clinical tools. 10 A cross-sectional area of the MN ≥ 10 mm ^2^ on ultrasound (US), measured just proximal to the pisiform, and electrodiagnostic findings of distal motor latency ≥ 4.2 ms and/or distal sensory latency ≥ 3.2 ms support the diagnosis.

Indications for surgical treatment are determined by symptom severity, response to conservative measures, and the potential for permanent nerve damage. The Royal College of Surgeons recommends CTR for patients with moderate or worsening symptoms despite conservative management, or for those presenting with sudden and severe symptoms. 11 Urgent surgery is indicated in cases of recent denervation with persistent sensory changes, rapid symptom progression, or high risk of irreversible nerve injury. Surgical intervention is also warranted in nonidiopathic CTS caused by space-occupying lesions, as well as in acute CTS secondary to trauma, infection, or hemorrhage. Early surgery may additionally be considered at the patient's request. A statistical evaluation identified five factors significantly associated with treatment response: age > 50 years, symptom duration > 10 months, constant paresthesia, coexisting stenosing flexor tenosynovitis (e.g., trigger finger [TF]), and a positive Phalen test within 30 seconds. 12 Patients presenting with more than three of these factors may require surgical intervention.

To avoid unsuccessful CTR, other neurological disorders that can mimic CTS such as polyneuropathy, radiculopathy, motor neuron disease, spondylotic myelopathy, syringomyelia, and multiple sclerosis, should be excluded preoperatively through consultation with neurology, neurosurgery, and rehabilitation medicine. 13

## Primary Carpal Tunnel Release

The skill and experience of the surgeon are essential in CTR, whether open or endoscopic, for recognizing anatomical variations and avoiding complications. Greater surgical volume and experience are associated with better postoperative outcomes. Among the different techniques for CTR, comparative assessments of factors such as incision length, operative time, postoperative pain, cost, and other related variables are summarized in Table 1 .

**Table 1:** Comparative assessment of various surgical techniques for carpal tunnel release

Surgical technique	Standard open CTR 14 15 16 17 18 19	Mini-open CTR 20 21 22 23 24 25 26 27 28	Endoscopic CTR 29 30 31 32 33 34 35 36 37	Minimally invasive CTR 38 39 40 41 42 43 44 45 46 47 48
Incision	3–5 cm	1–2 cm	<1 cm	3–5 mm
Symptom relief and patient satisfaction	Excellent long-term outcomes, consistent decompression	Comparable to open CTR; associated with less pain and faster recovery	Faster recovery, earlier patient satisfaction	Fastest recovery, high satisfaction, comparable outcomes
Operative time	15–40 min	12–15 min	25–45 min	< 10 min
Overall complication rate	10%; scar tenderness 19–61%	8–9%; minimal scar tenderness	5–6%; comparable to mini-open CTR; mostly transient neurapraxia	<2%; very low rates, (limited data)
Pillar pain	11–25%	<10%	<15%	< 1% (limited data)
Transient nerve injury	∼2%	∼2%	∼7% (about threefold higher than open CTR)	∼1% (ultrasound guidance may reduce risk of nerve injury)
Grip and pinch strength	Modest early decline, normalized within 24 wk	Mild early decline, slower recovery than endoscopic CTR	Mild early decline, fastest initial recovery	Comparable to, or better than, mini-open CTR
Time to return to work/daily activity	7–14 d/3–6 wk	5–7 d/2–4 wk	3–5 d/1.5–3 wk	2–4 d/1–2 wk
Reoperation rate	1–1.5% (very rare)	0.5–1%	1–2.3%	<1% (limited data)
Procedure cost/total cost	$1,000–2,600/$8,000–9000	Comparable to open CTR	$1600–3,300/$8,000–9,000	$≈3,000/$≈4,000
Summary	Gold standard for long-term outcomes and decompression quality, despite more extensive dissection and longer recovery	Balanced efficacy and reduced invasiveness, with quicker recovery and minimal scar formation, although outcomes vary depending on the surgeon's experience	Excellent in terms of early return to activity and patient satisfaction but associated with a higher risk of transient nerve injury due to blind trocar insertion	Excellent potential for rapid recovery and fewer complications but limited evidence and a need for further clinical data

Abbreviation: CTR, carpal tunnel release.

### Open Carpal Tunnel Release

This technique provides direct visualization of the MN and the carpal tunnel, facilitating complete release, allowing treatment of concurrent pathologies, and reducing the risk of incomplete decompression.

**Fig. 1:**
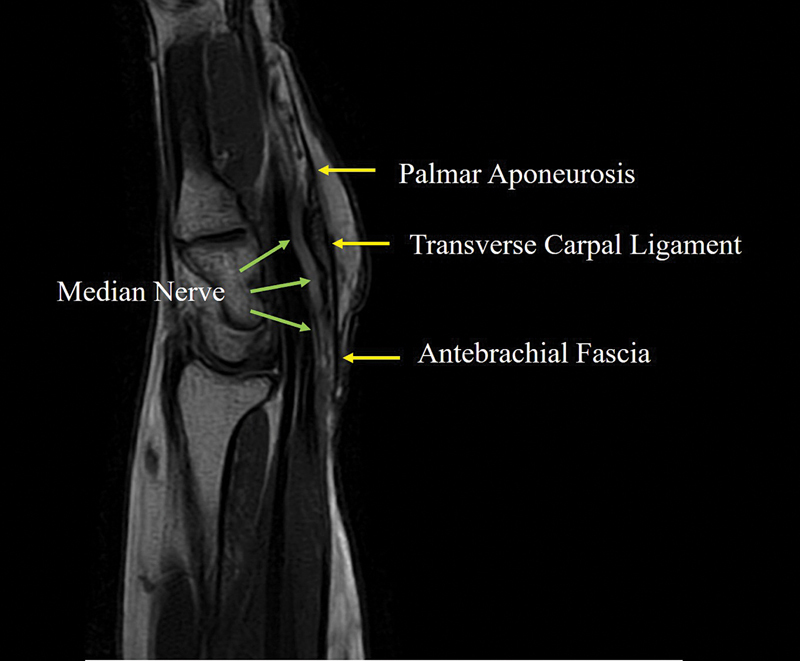
Sagittal MRI scan in severe carpal tunnel syndrome. The palmar boundary of the carpal tunnel is formed by three continuous segments of the flexor retinaculum: (1) a thin proximal segment composed of thickened deep antebrachial fascia of the forearm, (2) the transverse carpal ligament, and (3) a distal portion composed of the palmar aponeurosis between the thenar and hypothenar muscles. MRI, magnetic resonance imaging.

The palmar boundary of the carpal tunnel consists of three continuous segments of the flexor retinaculum: a thin proximal segment formed by thickened deep investing fascia of the forearm; the transverse carpal ligament (TCL); and the distal portion, composed of an aponeurosis between the thenar and hypothenar muscles ( Fig. 1 ). Anatomically, MN compression most commonly occurs at two locations within the wrist. 14 The first is at the proximal edge of the TCL. The second is at the level of the hook of the hamate, where the carpal tunnel is narrowest, measuring approximately 20 mm in width compared with 24 mm proximally and 25 mm distally.

**Fig. 2:**
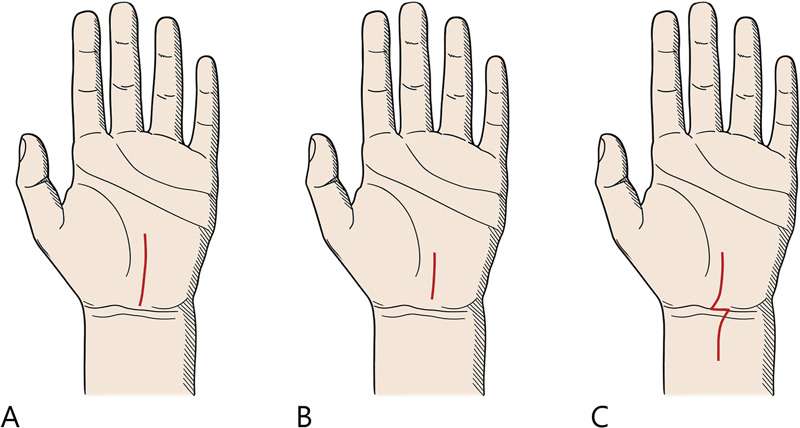
Various incisions for carpal tunnel release. ( **A** ) Classical standard incision along the longitudinal axis of the palm. ( **B** ) Modified short incision by the senior author (S.H.W.) to reduce scar-related complications. ( **C** ) Extended incision by the senior author (S.H.W.), used in recurrent or complex cases requiring wider exposure.

The classic open CTR incision is a curved longitudinal incision that follows the thenar crease and crosses the wrist crease obliquely in an ulnar direction. It is typically aligned with the long axis of a flexed ring finger or placed just ulnar to the palmaris longus tendon ( Fig. 2 ). 15 Although incisions may vary in length and location, the senior author (S.H.W.) prefers shorter incisions of approximately 2 to 3 cm. In cases of recurrent CTS or concurrent pathologies, the incision may be extended in a Z-plasty pattern, with the central limb placed along the wrist crease. Optimizing incision length and placement is an important consideration for reducing intraoperative and postoperative complications in CTR. 16

Cadaveric studies have also demonstrated that releasing the TCL alone may leave residual pressures exceeding 30 mm Hg beneath the distal forearm fascia. 17 Release of the distal 3 cm of the antebrachial fascia directly proximal to the TCL has been recommended, as originally described by Phalen in 1972. 18 Therefore, the approach most commonly pursued at our institution is CTR with release of the 3 cm of antebrachial fascia proximal to the TCL, together with the palmar fascia and the transverse fibers connecting the thenar and hypothenar fascia located distal to the flexor retinaculum. 19 Using this method, referred to the Short-Incision Extended Decompression Technique at our institution, a 2- to 3-cm skin incision provides a sufficiently wide decompression, and the recurrence rate has remained as low as 0.1%. While this approach improves the reliability of decompression, it carries the risk of scar tenderness or pillar pain and may lead to delayed functional recovery in the early phase due to the comparatively longer skin incision than in endoscopic or mini-open techniques.

### Mini-Open Carpal Tunnel Release

This technique was developed as an alternative to traditional open methods to reduce the risk of scar tenderness, pillar pain, and delayed functional recovery in the early postoperative phase. In 1994, a mid-palm incision of approximately 1.5 to 2.0 cm was first described for carpal tunnel decompression. 20 Since then, various modifications have been proposed, aiming to further shorten the incision either in the mid-palm, 21 22 23 24 at the wrist crease, 25 26 27 or at both sites. 28

### Endoscopic Carpal Tunnel Release

Since the late 1980s, endoscopic CTR has attracted considerable interest, 29 30 being associated with faster functional recovery and reduced scar tenderness and pillar pain. In a 1992 study, the complication rate was 6% in the one-portal group and 5% in the two-portal group. 31 During the early adoption of this technique, complications such as tendon ruptures and injuries to vessels and to nerves, including the digital, median, and even ulnar nerves, were reported. With increasing clinical experience, however, endoscopic CTR has demonstrated efficacy comparable to open CTR. 32

Endoscopic CTR may offer advantages in terms of early patient satisfaction and quicker return to work. However, no conclusive evidence supports its long-term superiority over open CTR. 33 Recent systematic reviews have shown no significant differences between the two techniques with respect to complication rates, local pain, mean pain scores, overall patient satisfaction, symptom severity, functional outcomes, or mean operative times. 34

Transient postoperative nerve injury occurs more frequently with endoscopic CTR, regardless of the number of portals used, although overall complication and reoperation rates remain comparable. 35 The intraoperative conversion rate from endoscopic to open CTR is approximately 1.02%, most often due to poor visualization from hypertrophic tenosynovitis or aberrant nerve anatomy. 36 Despite the absence of clear clinical superiority, the use of endoscopic CTR has steadily increased. A retrospective cohort study of more than two million cases between 2010 and 2021 showed that the proportion of endoscopic CTR rose from 15.7 to 26.1% during this period. 37 Independent predictors of undergoing endoscopic CTR rather than open CTR included younger age, female sex, fewer comorbidities, and geographical variation. By 2021, more than one-fourth of CTR procedures in the United States were performed endoscopically, underscoring its growing popularity.

### Ultrasound-Guided Carpal Tunnel Release

Technical advances in ultrasonography, with higher spatial resolution and excellent depiction of anatomical landmarks, have enabled US-guided percutaneous CTR with improved clinical accuracy and reliability. 38 39 Using axial US images of the carpal tunnel, a “safe zone” was defined between vertical lines drawn from the ulnar margin of the MN to the radial margin of the ulnar artery. 40 One or two puncture sites smaller than 5 mm are made on the palm and/or distal wrist. 41 42 A multicenter pragmatic study of long-term outcomes demonstrated significant and clinically meaningful improvements in symptoms and function, which were maintained at 1-year follow-up. However, further comparative studies are required to establish therapeutic efficacy. 43 44

### Other Minimally Invasive Techniques of Carpal Tunnel Release

Thread CTR involves passing a piece of thread percutaneously under US visualization, rather than performing a traditional surgical release with a knife. 45 This procedure requires only one needle entry point at the wrist and one exit point in the palm and can be performed safely and effectively under local anesthesia in a clinic setting.

Another minimally invasive technique uses a hook knife introduced through a small transverse incision just above the wrist crease. 46 An experienced surgeon can perform this procedure in an outpatient clinic with local lidocaine infiltration and a proximal tourniquet. While it offers advantages such as minimal incisions, quicker return to work, and reduced scar tenderness and pain, it is associated with a steep learning curve.

However, these minimally invasive techniques have limited effectiveness in cases of secondary or recurrent CTS. Furthermore, the incidence of iatrogenic injury to the so-called “million-dollar nerve,” the thenar muscular branch of the MN, 47 accounts for 3.6% of all major reported complications after CTR. 48 Reliance solely on indirect visualization through US may increase the risk of inadvertent injury to this nerve and other nearby structures, including the digital nerves and the ulnar artery and nerve.

## Complications of Carpal Tunnel Release

### Pillar Pain

Pillar pain after CTR typically arises in the critical pillar rectangle of the palm, where the palmar skin and subcutaneous tissue are less mobile. 49 Several factors have been implicated in its development, including widening of the carpal arch, periostitis of the hamate and scaphoid tubercles, intrinsic muscle pain from tension in the released TCL, and transection of small nerve fibers. 50 However, the exact cause remains unclear, with proposed mechanisms involving ligamentous or muscular changes in the carpal arch, neurogenic factors, and edema. 51 A recent systematic review found no significant reduction in pillar pain following endoscopic CTR, flexor retinaculum lengthening, short-incision techniques, or illuminated knife methods compared with open CTR. 52 Standard open CTR may be associated with a longer duration of pillar pain, often lasting 3 to 6 months postoperatively; however, most cases resolve by 6 months.

The multifactorial nature of pillar pain, including potential neurogenic components from transection of small nerve endings, underscores the importance of managing wound contraction through hand therapy. Such therapy promotes nerve regeneration and helps alleviate pillar pain. Because pillar pain is a common cause of delayed recovery and prolonged time away from work, effective management strategies are essential. These may include restricted hand use, splinting, symptomatic superficial hot and cold compression, or massage. In addition, extracorporeal shock wave therapy has been reported as a safe, noninvasive treatment option. 53 54

### Recalcitrant Carpal Tunnel Syndrome

Both endoscopic and open CTR are considered safe and effective for the management of CTS. However, revision surgery is required in approximately 1 to 5% of patients, most commonly for failed relief of the initial symptoms, recurrence of symptoms after a symptom-free interval, or the development of new symptoms. 55 56 57 58 For appropriate management, it is essential to differentiate symptoms as recurrent, persistent or new for revision surgery.

A large cohort study reported that the cumulative incidence of revision was 1.06% at 5 years and 1.59% at 10 years. 59 Endoscopic CTR has been associated with a significantly higher hazard of revision, most commonly due to symptom recurrence, which accounts for 58.7% of revisions. Reconstitution of the TCL was more common after endoscopic CTR compared with open CTR. In addition, incomplete release of the TCL occurred in 13.9% of cases, also more often after endoscopic CTR. When incomplete release was identified, the distal portion of the TCL was the segment most often left unreleased across both techniques. However, an intact proximal TCL was observed more commonly after open CTR, whereas an intact distal portion was more commonly seen after endoscopic CTR. Current evidence, however, does not indicate a significant increase in the rate of permanent nerve injury associated with endoscopic CTR. 32 60 61

#### Recurrent Carpal Tunnel Syndrome

**Supplementary Video S1** A 61-year-old woman had undergone carpal tunnel release at another clinic 4 months earlier. She reported initial postoperative improvement, but subsequently experienced worsening numbness and tingling in the hand, which prompted presentation for further evaluation. Using the previous incision, a reconstituted portion of the transverse carpal ligament was identified and reopened together with the associated scar tissue. In addition, just distal to the wrist crease, intact ligamentous structures that had not been released during the prior surgery were observed, necessitating a proximal extension of the incision across the wrist crease. Both the transverse carpal ligament and the forearm fascia were subsequently divided completely. The median nerve was found to be severely adherent to the radial wall of the carpal tunnel, and external neurolysis was performed. The patient's symptoms were immediately relieved postoperatively.


**Fig. 3:**
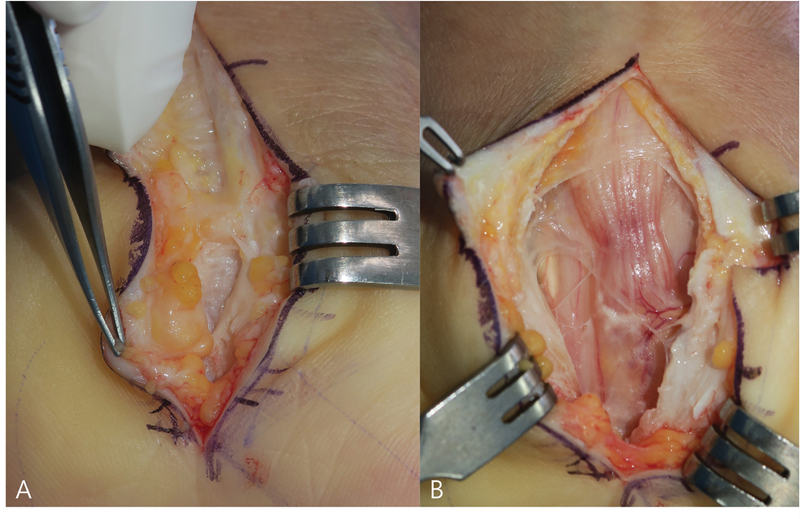
A 66-year-old woman presented with thenar atrophy, numbness, and pain in the left hand, 11 years after an initial carpal tunnel release at another hospital. ( **A** ) Reconstituted transverse carpal ligament. ( **B** ) Severe compression of the median nerve just proximal to the wrist crease was confirmed through an extended palmar and wrist incision. Symptoms improved after complete re-release of the transverse carpal ligament.

Recurrent CTS is defined as the reappearance of symptoms at least 6 months after the initial surgery. The most common cause is perineural adhesion, usually due to excessive scarring, which may require neurolysis, nerve wrapping, or local flap coverage of the MN. The second cause is reconstitution of the TCL, which can be managed by secondary release of the ligament. Finally, secondary compression may also occur due to tenosynovitis, postoperative infection, or hematoma ( Fig. 3 , Supplementary Video S1 , available in online version only).

#### Persistent Carpal Tunnel Syndrome

**Supplementary Video S2** Experimental demonstration showing that incomplete release of the transverse carpal ligament may exacerbate median nerve compression. A wide rubber band was used to simulate the ligament and yellow clay to represent the median nerve. The video illustrates that partial release, particularly when opened halfway, produces greater compression of the clay than either no release or complete release.


Persistent CTS refers to symptoms that continue without resolution after the primary surgery. These may result from incomplete release, irreversible nerve pathology, or an incorrect diagnosis in the presence of concomitant proximal neural compression. In cases of incomplete release, the most distal portion of the TCL and the proximal antebrachial fascia are the most likely sites of ongoing compression. This residual narrowing may exacerbate symptoms by producing even more severe compression from the remnant TCL ( Supplementary Video S2 , available in online version only). Assessment with US or MRI is helpful to confirm incomplete release, and secondary release of the remnant TCL can dramatically improve symptoms.

Compression of the MN at a more proximal site, such as in the forearm (pronator syndrome) or in the cervical spine, may lead to misdiagnosis as CTS. In such cases, persistent symptoms after CTR may be explained by a “double-crush” syndrome. This syndrome is defined as multiple compression points along a single peripheral nerve, which are thought to increase vulnerability to axonal damage. A retrospective review in 1985 highlighted the “double-crush” phenomenon, demonstrating its influence on both the clinical manifestations of CTS and surgical outcomes. Cervical spine abnormalities, most commonly narrowing of the C5–6 or C6–7 disc space, were present in 81% of patients with suboptimal results after CTR. 62 In cases with symptomatic compression at both sites, performing CTR first is considered a reasonable approach before more invasive cervical spine operations are undertaken. 63

#### New Symptoms after Carpal Tunnel Release

**Fig. 4:**
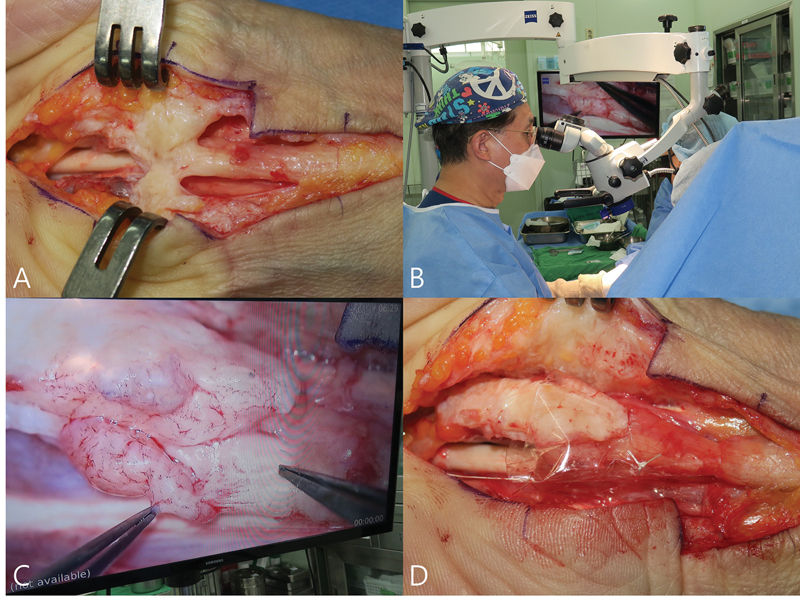
A 56-year-old woman had undergone two carpal tunnel release operations at a local clinic within a 3-week interval, 10 months earlier, for right carpal tunnel syndrome. Postoperatively, she developed severe coldness, tightness, fingertip pain, and thenar atrophy, resulting in disabling numbness and sleep disturbance. At 10 months after the initial surgeries, revision surgery was performed. ( **A** ) Severe parenchymal injury of the median nerve with neuroma formation and dense adhesions to the radial and ulnar walls of the carpal tunnel. ( **B, C** ) Neurolysis under the microscope. ( **D** ) Median nerve wrapping with an antiadhesive material. At 2-year follow-up, numbness had improved slightly and daily functioning became more manageable, although sensory loss in the index finger persisted. The DASH score improved from 65 to 45, but pinch and grip strength showed no significant change.

Completely new symptoms, such as numbness or paresthesia in previously unaffected areas or new weakness of the thenar muscles after CTR, suggest the possibility of iatrogenic nerve injury ( Fig. 4 ). In such cases, patient complaints should not be ignored or managed expectantly; instead, early reexploration and meticulous microsurgical nerve repair are recommended.

Other problems, including pillar pain, latent TF, and morning stiffness of the finger joints, are also frequently encountered. Therefore, preoperative evaluation with a detailed history of concurrent discomfort and pain, as well as clear explanation of the expected postoperative course including the potential for pain and slow progress, particularly in extremely severe cases, is essential. This enables both the surgeon and the patient to distinguish true new symptoms from expected postoperative findings.

### Revisional Surgery

An appropriate approach to failed CTR begins with a thorough medical history, clinical examination, electrodiagnostic testing, ultrasonography, and magnetic resonance (MR) neurography, to precisely determine the cause of persistent or recurrent symptoms and the indication for revision surgery. Conservative management may provide symptomatic relief through scar management, splinting, and nerve-gliding exercises but is appropriate only in the absence of suspected nerve injury or concurrent neuropathy.

In most other cases, management of failed CTR requires revision surgery, which may include redo release of the TCL, external neurolysis, and the use of various flaps to cover a scarred or damaged nerve. A hypothenar fat pad ﬂap based on perforators of the ulnar artery is the most frequently recommended. 64 A systematic meta-analysis suggested that decompression with vascularized flap coverage achieves a higher success rate of 86% compared with 75% for simple repeat decompression. 65 Other adjunctive options after neurolysis include vein wrapping, biologic wrapping with absorbable semipermeable materials, or synthetic collagen wraps, which provide a mechanical barrier around a scarred nerve or one with an epineural injury.

Symptomatic improvement following revision surgery was slightly better after open CTR, at 90%, than after endoscopic CTR, at 76%. 66 However, the rate of complete symptom relief after revision surgery was similar between the two approaches. In cases of inaccurate diagnosis, concomitant nerve pathologies, or insidious recurrence, outcomes can improve with appropriate treatment. However, iatrogenic nerve injury or severe scarring around the nerve may result in permanent symptoms of paresthesia, pain, or weakness. In other words, the surgical outcomes of revision surgery largely depend on the integrity of the remaining MN parenchyma.

## Nonidiopathic Carpal Tunnel Syndrome

Apart from idiopathic CTS, various systemic or local factors can alter the balance between the contents and capacity of the carpal canal, leading to elevated tunnel pressure and subsequent clinical symptoms. Well-known systemic causes include diabetes and pregnancy. Local causes include space-occupying lesions (SOL), inflammation, trauma, and anatomical anomalies. Although endoscopic or minimally invasive surgery to release the TCL has become increasingly common, such approaches may fail to identify underlying local causes of compression, and symptoms may not subside.

### Space-Occupying Lesions

**Fig. 5:**
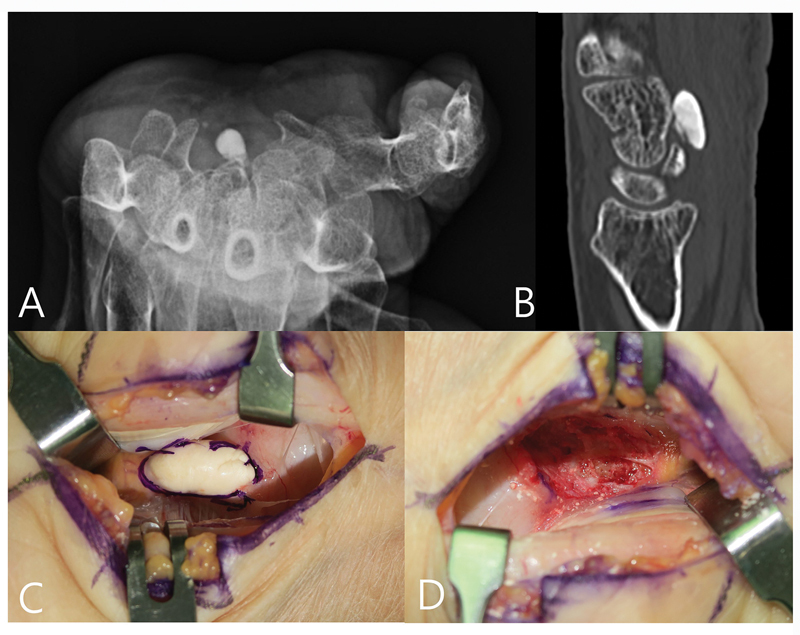
A 77-year-old woman presented with a 3-year history of severe numbness confined to the left hand with thenar atrophy. ( **A** ) Carpal tunnel view X-ray showing an oval calcification. ( **B** ) Sagittal CT scan confirming the lesion located anterior to the hamate. ( **C** ) Intraoperative photograph showing the calcific mass. ( **D** ) Operative field after carpal tunnel release with resection of the calcific mass. The patient's symptoms improved postoperatively. CT, computed tomography.

The SOLs such as intracanal tumoral calcinosis ( Fig. 5 ), tophaceous gout, schwannoma, intraneural hemangioma, ganglion, lipoma, fibroma of the tendon sheath, and even synovial sarcoma can contribute to CTS either by exerting direct pressure on the MN or by causing inflammation within the tunnel. 67 68 69 In patients with atypical progression of CTS, in those with unilateral CTS, or in cases of recurrence after endoscopic release, plain radiography with a carpal tunnel view, ultrasonography, or MRI is mandatory to evaluate for SOL.

### Tenosynovitis

**Fig. 6:**
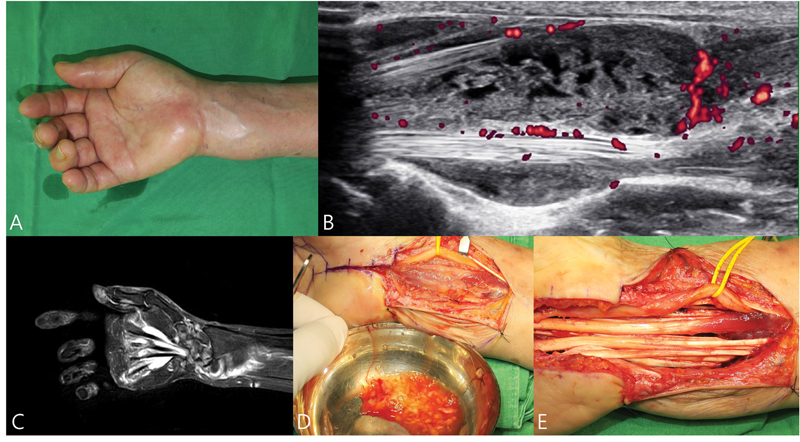
A 65-year-old woman developed severe swelling, pain, and limited flexion 6 months after right carpal tunnel release. ( **A** ) Clinical photograph showing marked swelling of the wrist. ( **B, C** ) Ultrasonography and MRI showing extensive flexor tenosynovitis with rice body–like signals and synovial thickening along the thumb and little finger. ( **D, E** ) Rice bodies and inflammatory fluid after tenosynovectomy. Biopsy revealed chronic granulomatous inflammation, and culture grew *Mycobacterium intracellulare* . MRI, magnetic resonance imaging.

Flexor tenosynovitis of the wrist caused by rheumatoid arthritis (RA) or tuberculosis (TB), can present symptoms similar to those of CTS ( Fig. 6 ). Its incidence varies depending on the underlying condition and the specific joint or bursa involved. This is particularly relevant in patients from TB-endemic areas and in those with atypical CTS combined with unilateral diffuse swelling of the volar wrist. 70 71 It is also an important consideration in patients with persistent symptoms after simple CTR. 70 71 In such cases, radiological confirmation with ultrasonography or MRI is mandatory, guided by the presenting symptoms.

Rice bodies are commonly associated with both RA and TB, but they are not pathognomonic. Therefore, if infection is suspected, definitive confirmation should be obtained intraoperatively using acid-fast bacilli stain, mycobacterial culture, immunohistochemistry, or polymerase chain reaction assay. Once infection is confirmed, appropriate antimycobacterial therapy should be administered for approximately 1 year. In patients with negative smear results, treatment should begin after histopathological confirmation, as culture and sensitivity results may be delayed by up to 6 weeks. 67

### Acute Wrist Trauma

Acute trauma to the wrist, such as a perilunate dislocation or distal radius fracture, can lead to functional sequelae including pain, stiffness, and neurological symptoms. When signs of MN compression are present, CTR is generally not performed because reduction of the dislocation or fracture usually alleviates the nerve symptoms. 72 Nevertheless, approximately 13% of perilunate injuries have been reported to be associated with CTS within 1 year of treatment. In addition, patients who underwent operative treatment were diagnosed with CTS about seven times more often and underwent CTR more than 10 times more often than those treated nonoperatively. 73

High-energy injuries in young patients are particularly likely to induce acute CTS. Distal radius fractures are strongly correlated with CTS when fracture translation exceeds 35% and in female patients younger than 48 years. 74 The incidence of acute CTS in the setting of distal radius fractures has been reported at approximately 5.4%, consistent with previous studies that range between 0.2 and 21.5%. 75 Aggravated symptoms may result from hemorrhage into the tunnel at the fracture site as well as splint positioning in significant wrist flexion. Surgical decompression and bony stabilization within 36 hours are recommended to protect and preserve nerve function. 76

### Anatomical Variation: Bifid Median Nerve and Persistent Median Artery

**Fig. 7:**
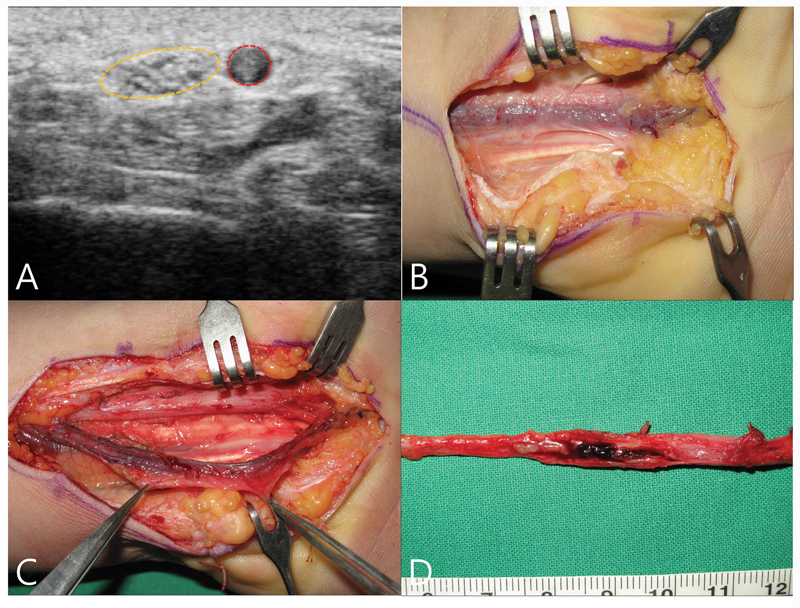
A 26-year-old man presented with a 1-week history of fingertip numbness and wrist pain, without trauma. Plain radiographs were unremarkable, while light-touch sensation was decreased in the median nerve territory. Tinel's sign and compression tests at the wrist were positive, but nerve conduction studies and electromyography were normal. ( **A** ) Ultrasonography showing a hypoechoic mass along the median nerve in the distal one-third of the forearm, without Doppler pulsation. ( **B** ) Intraoperative view of a thrombosed vessel running along the median nerve. ( **C** ) Microsurgical dissection of the median artery with resection of approximately 10 cm from the distal forearm. ( **D** ) The lumen of the resected artery completely filled with thrombus. Histopathological examination revealed organizing thrombus within the arterial wall. The patient's symptoms of numbness and pain resolved immediately after surgery.

After the eighth week of gestation, the median artery undergoes regression with the subsequent development of the radial and ulnar arteries. A persistent median artery (PMA) is an uncommon condition, present in approximately 3.7% of wrists and independent of ethnicity, age, sex, or occupation. 77 PMA and bifid median nerve (BMN) frequently co-occur, 78 but BMN alone is not considered an independent risk factor for the development of CTS. 79 Although rare, thrombosis of a PMA can lead to acute CTS. This unusual presentation typically occurs in young adults following blunt trauma to a unilateral wrist ( Fig. 7 ). The sudden onset of numbness in the MN distribution, accompanied by finger pain, may serve as a diagnostic clue. Prior to surgery, ultrasonography and MR angiography should be performed to confirm the diagnosis. Surgical treatment, consisting of resection of the thrombosed vessel and decompression, effectively relieves symptoms. 80 81 82

## Adjunct Hand Surgery with Carpal Tunnel Release

### Opponensplasty

Even in severe CTS, CTR alone provides symptomatic improvement in functions such as power grip, key pinch, tripod pinch, index–thumb pulp pinch, and thumb opposition. Electrophysiological studies have also demonstrated improvements in motor and sensory amplitudes, distal motor latencies, and sensory conduction velocities. 8 In patients with severe CTS and thenar atrophy, the presence of a detectable compound muscle action potential of the abductor pollicis brevis on electrophysiological testing suggests that thumb opposition may recover after CTR alone. 83 For this reason, routine simultaneous opponensplasty is not recommended and should be reserved for selected cases.

Nevertheless, recovery of thumb opposition remains highly unpredictable because of the chronicity of MN compression and longstanding thenar muscle loss. A subsequent or simultaneous opponensplasty may still be necessary to improve thumb function. Since the introduction of the Camitz procedure in 1929, 84 numerous modifications have been described to address drawbacks such as poor rotation during thumb opposition, tendon bowstringing, flexion of the metacarpophalangeal joint, and painful palmar scarring. 85 86 87 88 89

**Fig. 8:**
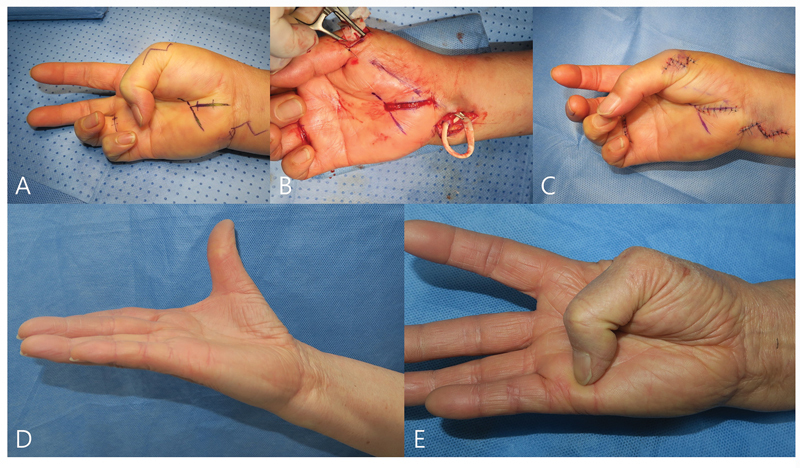
A 60-year-old woman presented with thenar atrophy and numbness of the median nerve–innervated digits and was diagnosed with extremely severe carpal tunnel syndrome based on nerve conduction velocity and electromyography findings. Primary opponensplasty was performed simultaneously with carpal tunnel release under wide-awake anesthesia. ( **A** ) Preoperative absence of thumb opposition. ( **B** ) Bunnell's opponensplasty performed by transferring the fourth FDS tendon to the thumb MP joint region via an FCU sling at the ulnar wrist. ( **C** ) Immediate postoperative restoration of thumb opposition. ( **D, E** ) At 4-year follow-up, both grip and pinch strength had recovered to the same level as the contralateral side. The patient reported marked improvement in chopstick use and button fastening, and the thumb, which could not be elevated preoperatively, showed full elevation. The DASH score improved from 19 preoperatively to 2 postoperatively. FCU, flexor carpi ulnaris; FDS, flexor digitorum superficialis.

A recent systematic review reported that the most commonly used tendon transfers were the PL, extensor indicis proprius (EIP), and flexor digitorum superficialis (FDS). 90 All transfers improved range of motion, pinch strength, and Kapandji scores. Complication rates of 19% with FDS and 12% with EIP transfers were mostly related to donor-site morbidity, whereas PL transfers had a complication rate of 6%, most often due to bowstringing. Among these various options, we particularly favor the use of the FDS as a donor. The use of the FDS as a donor provides an appropriate distribution of tension across abduction, flexion, and pronation, making it well-suited for opponensplasty ( Fig. 8 ). 91 92 93

### Neurolysis

A meta-analysis suggested that patients who underwent neurolysis reported worse global outcomes compared with those who did not. 94 However, in revision cases, external neurolysis (nerve mobilization) is generally advocated, as these cases almost invariably demonstrate extensive scarring, adhesions, and constriction of the MN by soft tissues within the carpal tunnel.

### Flexor Tenosynovectomy

Histopathological studies of the flexor tenosynovium in primary idiopathic CTS have demonstrated predominantly noninflammatory changes and have failed to establish an association between CTS and systemic conditions such as obesity, diabetes, or thyroid disease. 95 Based on the assumption that chronic flexor tenosynovitis could be an etiologic factor in idiopathic CTS, flexor tenosynovectomy has been performed as an adjunct to routine CTR. However, a meta-analysis demonstrated that this procedure provides no additional benefit and should not be performed routinely in primary CTS. 96

Flexor tenosynovectomy may still be considered in recurrent or secondary CTS. Histopathological findings of the flexor tenosynovium in recurrent cases are generally similar to those in primary idiopathic CTS, with a slightly higher prevalence of amyloid deposition reported in older men. However, no patients have been reported to develop systemic amyloidosis. Therefore, routine biopsy of the flexor tenosynovium in idiopathic or recurrent CTS is not routinely recommended. 97

### Trigger Finger Release

TF occurs at higher rates in extremities affected by CTS, supporting the hypothesis that an inflammatory process may contribute to its development. 98 The flexor tendons pass through the carpal tunnel and continue distally to the finger pulleys; thus, tendon and pulley mechanics may influence each other during wrist or finger motion and even after CTR. For example, A1 pulley release may alter tendon excursion forces, thereby increasing the likelihood of CTS development. 99

Conversely, a prospective study demonstrated that CTR itself is a significant risk factor for the onset or aggravation of TF. 100 CTR alters the environment inside and adjacent to the carpal tunnel in the early postoperative period, causing anterior displacement of the flexor tendons at the wrist after division of the TCL. This bowstringing effect increases the attack angle of the flexor tendons against the A1 pulley, thereby augmenting frictional and compressive forces at the tendon–pulley interface. The increased mechanical load, along with deterioration of the boundary lubrication mechanism, may precipitate the development of TF.

Because of this interplay, hand surgeons should routinely evaluate for both TF and CTS, even in the absence of patient complaints. Moreover, when TF is present, it should be managed more proactively at the time of CTR, either through concomitant surgical release of the A1 pulley or steroid injection in cases of early TF. 101

## Conclusions

CTS has emerged as an increasingly prevalent condition worldwide, contributing to substantial socioeconomic burdens across healthcare systems. The success of surgical intervention, however, remains highly dependent on accurate diagnosis established through meticulous history taking, comprehensive physical examination, and systematic exclusion of alternative diagnoses. When appropriate patient selection and diagnostic criteria are met, CTR consistently yields high patient satisfaction rates, establishing it as one of the most successful procedures in hand surgery.

Various surgical approaches, including open, endoscopic, and mini-open techniques, each present unique advantages and limitations. However, clinical evidence suggests that the choice of surgical method based on individual patient characteristics does not significantly affect overall outcomes. Instead, patient-specific considerations such as socioeconomic status, occupational requirements, insurance coverage, comorbid medical conditions, and identification of secondary causes should guide surgical decision-making, together with surgeon experience and preference. These factors collectively contribute to treatment individualization without compromising therapeutic efficacy.

Nevertheless, the adoption of experimental or emerging surgical techniques should be approached with caution. The widespread implementation of new procedures requires robust clinical evidence, long-term outcome data, and comprehensive safety profiles drawn from the scientific literature. Equally, the safe application of novel surgical approaches depends on the surgeon's own profound understanding of carpal tunnel anatomy, acquired through extensive clinical experience. This principle ensures that innovation proceeds responsibly, minimizing complications and optimizing patient outcomes while advancing the field through evidence-based practice.
